# Asthma diagnosis and treatment – 1026. Long term twice weekly dose of methotrextate in steroid dependent chronic allergic diseases

**DOI:** 10.1186/1939-4551-6-S1-P25

**Published:** 2013-04-23

**Authors:** Suman Kumar, Poonam Kumar

**Affiliations:** 1 Allergy and Immunology, Ent and Allergy Centre, Panchkula, India; 2 Respiratory Diseases and Clinical Immunology Society, India

## Background

The purpose of this review is to characterize the type of patient who may benefit from alternate, nonsteroidal agents and to examine the current evidence behind their use.

## Methods

Evaluated the use of oral or IM low-dose methotrexate vs placebo in steroid-dependent asthma patients using either parallel or crossover design. 100 patients enrolled in study attending the ent and allergy centre (India)panchkula patients were on long term steroid therapy for more than 1 years. Age of patients between 30 to 50 years. Study comparing methotrexate, 10 mg weekly orally, or placebo.

## Results

It was concluded that methotrexate allowed a good amount of reduction in oral corticosteroid compared to patients receiving placebo. The benefit of using methotrextate were very promising as compared to side effects of corticosteroids in steroid depoendent allergy diseases however, and should be balanced against the potential for side-effects associated with the use of methotrexate.

## Conclusions

With low dose weekly therapy of metotrextate we can reduce the side effects of prolonged steroids have suggested that methotrextate may have steroid-sparing benefits coupled to generally mild adverse events; although adverse effects were not of a serious nature they were observed in up to one-third of patients. Rare but potentially life-threatening adverse effects involving the pulmonary, hepatic and haematological systems remain of particular concern. methotrextate should therefore be considered as an adjunct to high dose inhaled STEROIDS in patients who require more than 10mg of predenisolone daily, and who experience severe and unacceptable steroid-related adverse effects. Treatment should only be initiated by physicians with experience in the use of the drug, and the relevant safety parameters should be closely monitored.

